# Gaining insight into the Clinical Practice Guideline development processes: qualitative study in a workshop to implement the GRADE proposal in Spain

**DOI:** 10.1186/1472-6963-6-138

**Published:** 2006-10-23

**Authors:** Carlos Calderón, Rafael Rotaeche, Arritxu Etxebarria, Mercé Marzo, Rosa Rico, Marta Barandiaran

**Affiliations:** 1 Alza Health Centre, Osakidetza-Basque Health Service, Avda Larratxo s/n, 20013 Donostia-San Sebastián, Spain; 2 District of Guipúzcoa Este-Ekialde, Osakidetza-Basque Health Service, Aristizabal 1, 20120 Hernani, Spain; 3 Primary Care Service, Catalan Health Institute, Gran Vía de las Corts Catalanes 587, 08007 Barcelona, Spain; 4 Health Technology Assessment Agency-Osteba, Health Department of the Basque Government, Donostia 1, 01010 Vitoria-Gasteiz, Spain; 5 Hernani Health Centre, Osakidetza-Basque Health Service, Aristizabal 1, 20120 Hernani, Spain

## Abstract

**Background:**

The GRADE method represents a new approach to grading the quality of evidence and strength of recommendations in the preparation of Clinical Practice Guidelines (CPG). In the context of a pilot study to assess the implementability of the system in Spain, we considered it relevant to gain an insight into the significance of the perceptions and attitudes expressed by the actual experts participating in the system try-out.

**Methods:**

Qualitative research with an ethnographic approach, through non-participant observation and focus groups within the context of a consensus workshop in which 19 CPG experts participated to evaluate the GRADE proposal using 12 evidence tables taken from hypertension, asthma and arthritis CPGs. The interventions were recorded, under a guarantee of confidentiality. The transcriptions and field notes were analyzed, based on a sociological discourse analysis model, and the provisional findings were re-sent to participants in order to improve their validity.

**Results:**

1) Certain problems over procedure and terminology hindered the acceptance of this new method as a common reference system for the preparation of CPGs. 2). A greater closeness to clinical practice was accompanied by concerns over value judgments and subjectivity, with a demand for greater explicitness in the consensus process. 3). The type of "evidence" on which the guidelines are based, how and by whom the evidence is prepared, and what the role of the different actors should be, all constitute unresolved concerns in the CPG preparation and implementation processes. 4). The grading process is not neutral: professional background, prior experience and the degree of leadership all condition the participants' input and interactions.

**Conclusion:**

The findings obtained allow the quantitative evaluation to be better interpreted and, in turn, go beyond the particularities of the GRADE method. Adaptation to the complexities of clinical practice, the need for carefully designed multi-disciplinary work and the reflexivity present in the CPG preparation process, all represent lines of debate that are necessary to improve the CPG quality in the Spanish health care sector.

## Background

The last few years have seen the publication of an ever-increasing number of Evidence Based Clinical Practice Guidelines (CPG) by different Scientific Societies and Health Care Services in Spain, adding to those guidelines already existing at an international level and aimed at presenting clinicians with some top quality, decision-making instruments.

However, the increase in number of CPGs available has also been accompanied by recommendations that do not always coincide, contributing to confusion amongst professionals and lack of guideline uptake. Consequently, Spain is also placing emphasis on CPG quality and on the different CPG appraisal methods and instruments [1-3].

In this context, and after observing the limitations of the pre-existing grading systems with regard to evidence appraisal and the development of CPG recommendations[4], the GRADE method constitutes an initiative in which the principal stakeholders are participating in order to reach a consensus on a new method to develop recommendations simply, explicitly and systematically.

Consequently, once the specific clinical issue has been identified by the working party, the new method comprises the following steps: 1) Assessment of the quality of evidence across studies for the issue in question, which is then summarized in evidence tables for each important outcome. 2) Selection of those outcomes considered critical to a decision, differentiating critical outcomes from other important outcomes that are not considered critical. 3). Judgment on the overall quality of evidence for these critical outcomes. 4). Balance between benefits and harms, and between net benefits and costs, and 5) Grading of the strength of recommendations according to four categories: "do it", "don't do it", "probably do it" and "probably don't do it" [5].

The interest in this initiative led us to evaluate the feasibility of the GRADE system in the particular context of Spain, by reproducing its initial pilot studies [6] through an actual trial to prepare recommendations based on material taken from national CPGs and supported by the original GRADE technical documents translated into Spanish [7].

For this purpose, 20 experts in grading the quality of evidence and/or in the preparation of CPGs were selected. After participating in a specific training workshop, these experts worked individually with 12 evidence tables on real questions obtained from asthma, hypertension and arthritis CPGs. The participants subsequently met to discuss the recommendations developed following the GRADE method, to assess the degree of concordance and to try and reach a consensus in this respect. Likewise, the participants completed a pre-designed questionnaire on the principal problems encountered when applying the new method. The results of this have already been published [8] and as it can be seen in the Table 2 they show some critical findings with regard to the expected such as the fact that only 10.5% of participants should "agree" that the "method is clear and simple to apply" or that 89.5% should "disagree" or "strongly disagree" that "with the GRADE method, subjective decisions are generally not required".

Beyond the conclusions derived from the quantitative analysis of concordance and to understand better the meaning and sense of the experts' opinions, it was considered of interest to study the actual debate and consensus-seeking process "from within" by making a qualitative study of the attitudes and perceptions of the professionals who had participated in the trial. Numerous studies and proposals confirm the possibilities offered by qualitative research in the health service area in general and in the EBM area in particular [9-12]. However, in the specific area of CPGs, the majority of the research studies carried out have focused on the barriers to the practical implementation of CPGs [13] and the limited amount of research work directed at the internal CPG development process has been carried out in other contexts [14,15].

Consequently, we decided to carry out a qualitative study in order to gain an insight into the significance of the reactions and attitudes expressed during the trial to assess the new GRADE proposal in the Spanish health care sector.

## Methods

### a) Context, participants and data collection

The ethnographic study [16] was carried out in the same premises as used for the two technical consensus meetings, with the attendance of 19 of the 20 professionals who were initially asked to participate (Table 1).

The first stage of the study involved non-participant observation, whereby two researchers attended the technical consensus meetings (TCM1 and TCM2) as observers; these meetings were recorded and the behavior, attitudes and communications lines generated were noted down. The researchers were previously introduced by one of the promoters and all the participants agreed to the researcher participation in the study. The research material also included reports, forms and the questionnaire model used.

Once the technical consensus meetings had finalized, two focus groups (FG1 and FG2) were created with the 9 and 10 participants from each of the sub-groups into which the 19 experts participating in the study had been divided, considering the group technique to be ideal for promoting debate and interaction. These focus group meetings lasted approximately 90 minutes until all the contributions from within each group had been exhausted. One of the researchers with experience in qualitative studies acted as a moderator, requesting prior authorization to record the meetings whilst guaranteeing that the information would be treated confidentially. A second researcher acted as an observer, noting down the most relevant attitudes. These notes were then completed by both researchers at the end of the meetings.

### b) Analysis and validation

An analysis was carried out on the transcriptions of the technical meetings and focus groups, on the actual live recordings and on the observations noted down and the rest of the material compiled by the researchers. Cognitive mapping [17] was used to select and classify the different categories and the sociological discourse analysis model [18] was used as a reference model for the text analysis, as it was considered important to relate the contents with the framework in which they were generated. The ethnographic approach was particularly useful in articulating the descriptive and analytical dimensions of the research and in configuring concepts and categories.

All the researchers took part in the analytical process and discussed the results. The repeated comparison with the empirical material compiled in the texts (recordings, transcriptions and field notes), in addition to the search for negative cases, led to some modifications to the initial approach. The results were subsequently sent to the participants for any possible further input, which was also assessed and included in the analysis, all of which was aimed at improving its validity. The text of the report translated into English was revised by the authors participating in the meetings and focus groups in order to ensure the accuracy of the translation of the verbatim.

## Results

**Figure 1:**
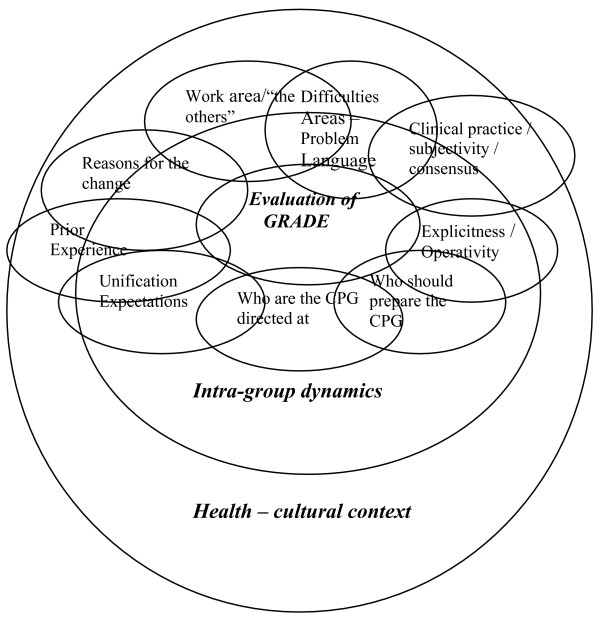
Dimensions and categories intervening in the GRADE method evaluation process.

Figure 1 summarizes the different dimensions and principal categories derived from the analysis of the results and their interrelation. As it can be observed, in practice the different factors overlap and act *as a whole *and this should be particularly taken into account in their assessment. The GRADE method was originally the object of debate but throughout the discussion and consensus process the experts repeatedly went and came back from the specific aspects of the method to other levels related to their experience as clinicians and health professionals in the GPC fields. This process was also grounded in the group dynamics and different experts' roles. Therefore, to facilitate the display, the results were classified into two basic lines: the first one closer to the particularities of the GRADE method and the second line directed at those findings emerged during the assessment of the new method but which pointed to more contextual factors.

### 1. Experiences in the use of the GRADE method

The analysis of the participants' perceptions helped understand certain responses to the quantitative questionnaire that were previously commented in the Introduction (Table 2) and that would otherwise be difficult to explain.

#### 1.a. General difficulties, problem areas and language

For both focus groups, the use of the new method was perceived to be difficult, confusing, not user-friendly, although it was recognized that acceptance gradually improved with use, indicating a certain haste and lack of time in the preparation and development of the workshop as possible conditioning factors.

"-...I thought the exercise was hard, long, the tables were difficult to understand, not very user friendly in general. That is, well, it's been difficult for me..."

-...At first, I thought it was very complex too... Then you start to get the hang of things and then you do go quicker..." (FG1)

"-...I think we skipped the steps that should have been done before the workshop...

-...as an instrument, I consider it to unfortunately be not very user-friendly" (FG2).

The degree of acceptance of the new method varied with the different exercises. The greatest confusion, observable in the examples of the diagnostic and prognostic tests, was related to the fact that these areas have not been well resolved in those grading systems already in use.

"-...the diagnostic tests drove me mad..." (FG1)

"-...As for me, due to the systematics of the work, I did the entire treatment base together and then, as I got used to it I said "well it wasn't' that bad", but then when that was completed I reached the diagnostics and I collapsed again.

-...I think that with the diagnostics we were all lost not just with the GRADE but with all of them, weren't we?"(FG2)

Likewise, the presence of some semantically unclear terms, perhaps as a result of their translation from the source language, was considered to be a possible factor of confusion:

-...I think that the term "probably do it" isn't very good because it's "you probably should do it. If you should do it, it can't be "probably".

-... if there's a really important issue, it's the language, the English. "(FG1)

-"...Here, isn't there a problem regarding Anglo Saxon culture and Latin culture? I look at it like a discussion, not because it's better or worse. Is this going to be any use to us here? Take for example, the problems we're having with the translation, with "trade off" "do it" "probably do it" we've never expressed it like that in Spanish..." (FG2)

It is striking that these negative impressions and limitations were not explicitly stated during the prior technical meetings, where the debates were centered on reaching a consensus on those aspects for which there had been less concordance. The leading role and the implicitly recognized authority of the more expert authorities was also more predominant at these meetings, and this was accentuated in the dialogue flow with the moderator and with the GRADE group representative.

#### 1.b. Greater closeness to clinical practice and concerns regarding subjectivity and consensus

The sequential nature of the method and the grading of the outcomes as more or less important caused different and occasionally conflicting impressions in the focus groups. On the one hand, favorable opinions were expressed for what was perceived as a greater closeness to clinical practice compared to prior methods which were declared to be too dogmatic in the interpretation and implementation of the EBM. However, on the other hand, concern was expressed over the subjectivity that the consensus factor may carry with it.

-"...I think that the good thing about it is that it focuses more on outcomes... however in practice I think that it's far too subjectivity weighted.... It follows therefore that, if the aim is to add subjectivity, such a complicated instrument is not really necessary "(FG1)

-"... I think that this is not leading to the consensus of old... the fact that certain subjective opinions are introduced in some items, I don't think it's necessarily a bad thing... these are contextual factors, values that we have and that can make us recommend or not recommend here, something that is in fact recommended in Japan. So we've introduced subjectivity? Well that's a good thing if it brings you closer... well I don't know ... I'm not afraid... any method of this type is like an improvement over the possible dogmatism we used to have..."(FG2)

The doubts were stronger with regard to the implementability of the recommendations or in the face of a limited amount of study evidence available for aspects perceived to be important in clinical practice.

"-...Do you think that this instrument measures implementability OK? What I mean is, is implementability valued at any point? That is the magnitude of the effect, whether it is accessible in my own particular area ... is this valued anywhere in this instrument?

-...The thing is, GRADE is more of a system for formulating recommendations rather than for adapting to local conditions, isn't it? FG2)

"- another thing that could happen to you is that there's nothing on the points you'd consider to be the most critical or the "best". So what do you do then? Do you change the most important to critical or not? because in actual fact you'd like to have information on something that's not actually there...

-...this is often what happens, you've got what you've got... is there a systematic review? All well and good. And, if there's not, there're three more or less acceptable tests and a good cohort study, and that's what there is, and you just have to make a combination based on what there is."(FG1)

The experiences immediately before the technical consensus meetings, with their corresponding dynamics of negotiations and concessions, probably contributed to emphasizing the concerns of subjectivity and value judgments.

"-...I'm also in favor of putting "do it" but if the patient says to me "look, you know this is a real pain for me and my family, and nothing'll happen if you don't do it, then "probably do it"

-.. I agree. That's the recommendation that I make in daily practice. That's why I put "do it". If you like, I'll back down and put "probably do it"...(TCM1)

"-I've put "do it" but I'm going to change it to "probably do it" (laughter)

- Yes, I've no objection to changing to "probably do it" either

- Neither have I.

-"Do it" if these are the conditions... I don't think there's any problem recommending "do it"..."(TCM2)

Faced with these problems, the need for greater explicitness was suggested, which in turn should be compatible with the instrument's ease of use.

"-...perhaps the way to resolve this is to be far more explicit. In other words, I've reached this conclusion because I've made the following considerations and write them down. When there's been a group discussion and a consensus has been reached, then explain how this consensus was reached, I think that this is a bit of what's still missing...

-...One thing about the explicitness we were talking about: I'm a bit cautious of this, because explicitness is complicated, what I mean is that, do we want to have everything so absolutely clear that we're not afraid of that situation of frustration? Then the tool becomes tremendously complicated, explicitness has to be kept within limits...(FG2).

#### 1.c. Who prepares the "evidence" and how

The tables model used by the GRADE system presupposes prior work on the primary studies (systematic reviews, clinical trails, cohort studies...). In some cases this was viewed favorably; however in others this generated concern over the quality and validity of the review process and over the selection of information. This concern was particularly marked amongst those participants with the greatest experience in critical reading.

"-...I know for sure that the evidence tables have made it easier for me, because I've based a lot of my work on the tables and these were graded. If I'd had to do it on the original work I'd never have been able to have sent this in before the deadline... So I think it's a very useful tool to have.

-...I don't know whether the studies that are missing are because they've not been included in the table or because they didn't really exist at the outset. I don't know anything about the quality of the evidence table either, because you don't know how they did it....

-...Do the epidemiologists have to prepare the guidelines for us? Or do we clinicians have to do them? In that case then,...

- The methodology has to be done by an expert, or at least the GRADE evidence table.

- I didn't want to go into that... because I think that you should do the GRADE evidence table with the studies, that is, I want to do it with the studies (M: so do I). The evidence table is OK but if constructing guidelines is like constructing buildings with something very difficult like bricks, which are the studies, I want to have the bricks." (FG1)

### 2. Beyond GRADE

The GRADE method assessment process revealed the existence of certain factors conditioning the experts' perceptions and which are not directly related to the particularities of the new method.

#### 2.a. Prior experience and expectations of working with a common system

In this respect, the experts' prior experience in the area of CPG preparation and grading appeared as an important factor, providing information on the expectations with which the new method was received and on the reasons behind some of the reservations in accepting it. In this way, the fact that the majority of the group had extensive experience in critical reading could be felt in the course of the technical meetings and in the more open evaluations in the focus groups.

-"...those of us who've prepared guidelines, we've already developed our model, and we're very biased towards doing it in that particular way... I think it's a matter to give some thought to... because we're all very biased by the way we're working in this field... you others have had much more basic work (in critical reading) and of course...(FG1)"

"-...I'd raise it too (referring to the grading in one of the tables) but... I'm very biased by the subject of the guidelines that we're...(laughter). I admit that that's the reason why...."(TCM2)

Due to the diversity of the existing grading systems, the advisability of joining forces around a common proposal was valued positively, although there was a call for more information on why changes are made and on the particularities of the process followed.

"-...as an instrument per se. I can see more advantages than disadvantages. Simply due to the fact that it exists, ... or that we all follow a more or less similar line, everyone working on guidelines worldwide and such, seems a good idea to me...

-...I think that that's very important, unification..." (FG2)

-"...Wouldn't it be easier to modify what's already done? ... try and adapt what there is and not make a radical change. I sometimes get the impression that this is a meeting of the "popes"

-...I think it's power, there's a lot of power here. I think they've seen that on the same clinical conditions, based on the same evidence, different recommendations come out... something's up here! And then no-one wants to give way ... they've got together and all this that appears very methodological ... they'll have to reach an agreement...FG2)

#### 2.b. Area of work and vision of "the others"

The possible influence of the participants' areas of work and background in the preparation and grading process constituted another aspect indicated in the focus groups and which goes beyond the particularities of the GRADE method.

"-...I do think that the reference of the people involved is important ... I think it's very important. On the one hand, those that have nothing to do with the clinic..." (FG1)

-"... I'd like to comment that, in the concordance study, it could be important to observe whether variability bears any relationship to profession, because the grading might possibly be different (everyone agrees) "(FG2)

However, in addition to their professional profiles and jobs, in the discourses of the expert participants there is also a characterization of "the others"; people that have to be counted on either in the preparation of future guidelines or those people that the guidelines are directed at.

In general these "others" were principally identified with specialist doctors or with GPs. The participation of these people was considered to be necessary but difficult and with different peculiarities, depending on the case.

"-...if we get them (the specialists) to do a critical reading of this, they'll disappear ... So, if you give them some good tables, you can tell them "fill in this questionnaire for me"...

-..that's true, for the outcomes. Perhaps the group would have to be first in order to allow the "hard core" to go and find things that respond to this and then we'd decide... because that's far more agreeable and closer to the day to day routine, the other is a pain in the neck"...

-...what happens is that these people have to be clinicians, because otherwise ...

-...we might possibly be giving all this too much thought, and the reader might happen to like being given the guidelines on a sheet. All he wants is to follow a recommendation, and he couldn't care less whether it's an "A" or anything else ...

-...that's quite true, when you present some guidelines, no-one's ever asked me about the letters.

- I've never been asked either." (GD1)

## Discussion

The results of this study respond to an initiative conditioned at the outset by the circumstances of the call to participate and the selection of participants for the specific purpose of making a technical appraisal of the GRADE initiative. The design of the qualitative part of the research work is therefore based on the above circumstances, a fact which has its limitations but also offers some possible advantages. On the one hand, not all the participants had the same prominence and, undoubtedly, the opinions of other experts and clinicians worthy of being heard, were left out. However, in turn, the possibility of studying the participants' reactions and impressions in their actual working environment, as experts, offered the advantage of less artificiality in carrying out the research.

With regard to the shortcomings experienced during the stage spent learning how this new method functioned, the lack of knowledge regarding how the tables were prepared, the language barriers, the greater difficulty perceived in the diagnostic and prognostic questions, and the concern expressed over the use of value judgments, the findings of this present study are of particular interest in helping to better explain the problems detected in the questionnaire completed by participants [8]. The input on specific aspects of the GRADE method should be taken into account with a view to the possible future implementation of this method in the Spanish health sector. Moreover, from a methodological point of view, the contributions lend continuity to earlier research work showing the potential for using qualitative methods as a "complement" at a later stage to questionnaire studies [19].

Furthermore, the input from this present qualitative research work can also be contemplated as a "differentiated" current [10], in the sense that it pays special attention to the significance and meaning of the attitudes and experiences expressed by the expert participants. In this sense, three principal lines of discussion can be considered and which go beyond the more technical or specific aspects of the GRADE method to reveal unresolved concerns in the GPC preparation process in general.

### I. The GRADE method and the tensions in the CPG preparation process: adaptation to the complexities of clinical practice

The pilot study to assess this new GRADE method confirms the inevitable need to relate the CPG discussion and preparation processes with the contexts in which they are generated [20,21].

The contents of the discourses and participant interaction reflect a reality marked by different *fields of tension*. Firstly, the perception of a certain divergence *between the evidence derived from the experimental studies on the one hand and clinical practice on the other*, underlining the non-linear nature of the relationship between the study design requirements and the complexity, dynamism and individuality present in health care work [22]. From this perspective, some of the concerns expressed in the previous section become understandable, such as the fact that certain "outcomes" considered important from a professional's point of view have not been the subject of a study or do not have the "quality" provided by experimental design; that the guidelines derived from a clinical test do not correspond to the specific characteristics of the patient requiring care; or the importance given to aspects such as external validity and implementability.

Consequently, and to the point that GRADE is perceived as being closer to those issues that professionals have to deal with in practice (outcome grading, process systematics, explicitness in the grading of recommendations), to a certain extent this new method is viewed favorably and appears to promote reflection. However, together with this greater closeness to reality, the need to express value judgments in order to finally reach a consensus with regard to the greater or lesser importance of the outcomes, provokes a marked *concern over subjectivity *and over the difficulty in expressing the said judgments in quantifiable tables.

The participants' desire to base professional practice on scientific criteria largely explains the opposition expressed. However, in turn, these fears and concerns also reflect the perplexity generated by the inevitable presence of *values *in a medium – such as the one represented by the CPGs – that is apparently exclusively reserved for the so-called *objectivity of facts*.

The forced separation of both dimensions (value judgments/facts that can be reduced to numbers) is also present in each of the different "repertoires" or facets (scientific, practical, political and procedural) present in the CPG preparation process [15].

In fact, the artificiality derived from concealing the value judgments and the frequent reduction of the evidence to something that is numerically quantifiable has already been questioned in the past in the light of what actually happens in clinical practice [23,24] and, in particular, in the numerous attempts to gain an insight into the barriers to CPG uptake and implementation in professional practice [25-27].

It therefore follows that the solutions to the said tensions should be sought not so much in the simplification of the instruments used to develop and evaluate the CPGs but in a more complete and thorough knowledge of the reality on which the CPGs are based and in a *dynamic and open adaptation of their design *to the requirements of this reality. In this respect, the decisions and assessments that professionals need to make in their daily practice should not be overlooked, so some excessively simplified response models ("do it" "probably do it") may be of limited benefit.

### II. By whom and for whom

A new area of tension is delimited by *the role corresponding to the different actors *involved in the CPG design and grading processes. In principle, both the GRADE method and those instruments already in place are based on the necessary participation of the clinician in the CPG development and dissemination process [28,29].

However, clinicians interested in the preparation and promotion of CPGs are first faced with a considerable number of studies published with insufficient guarantees of rigor and impartiality. They are thus forced to carry out the arduous task of systematically reviewing the biomedical literature in order to "separate the grain from the straw" [3]. This work, apart from requiring training and experience, tends to be given to "methodologists" or specialist technicians; the documentation prepared on this by NICE [29] possibly provides the greatest details of the tasks corresponding to each member of the CPG preparation group.

In our case, the display of mistrust for secondary data and the demand to access the original studies (the "bricks") by some of the participants in the research study, and the references to the "burden" that involvement in the mentioned critical reading tasks represents for many clinicians, would corroborate the importance of the tensions derived from the distribution of tasks in the CPG development process.

Certain authors have interpreted this tendency to divide the work as a possible paradigm shift according to which medical practice is moving from a more autonomous and individualized model to a more standardized model that depends on the criteria established by non-clinical professionals such as epidemiologists or bio-statisticians [30]. The concerns arising in this respect in this study paint a reality that is less dichotomic although it is undoubtedly sensitive to the said issues.

On the one hand, the majority of participants had worked in multidisciplinary groups to prepare CPGs and were aware of the advantages of doing so, both with regard to the distribution of tasks as well as the input from different professional perspectives. However, in turn, and probably as a result of their experience in critical reading, a marked prejudice towards the risks of misrepresenting scientific information can be inferred from their discourses. These risks are perceived to be greater as access to the original sources becomes more distant.

The greater degree of "scientific" authority that direct access to the data constituting the "evidence" confers on the "methodologists" [15] would help explain the concerns and mistrust put forward in the debate on the GRADE method by the clinicians in the face of possible irregularities derived from the "division of tasks". Clinicians, who in this case, had experience as authors of CPGs and were also witnesses to the difficult incorporation of their colleagues into this type of work.

Consequently, in the CPG development, in addition to the necessary *multidisciplinary collaboration*, it is also necessary to guarantee that the process is carried out on the basis of some common criteria of *rigor and transparency *right from the initial selection stages and secondary preparation of information up to the final dissemination of the recommendations.

The *detailed description of the functions and tasks *corresponding to the different members (epidemiologists, doctors, nurses, patients) of the CPG preparation groups [29] constitutes an unquestionable aid in this respect. However, greater attention should also be paid to the *specific requirements and risks of the intercommunications process based on the type of participant*. In our case, the "methodologists" and "clinicians" were the ones to reveal the "tensions" that appear to affect their inter-relations. Therefore, the debate on the different roles and task distribution dynamics of the CPG development process still remains open.

### III. Rigor and reflexivity: the internal environment

As indicated in the Results section, the different categories and dimensions analyzed interact through *intra-group dynamics*, an aspect which should be considered with particular interest from the point of view of CPG rigor and quality.

The "non neutral" component present in the attitudes shown by the different participants in the GRADE method pilot test is in line with earlier findings [14] and, in fact, the role played by the debate and consensus process in the final quality of the CPGs has not been overlooked by the experts in their attempts to standardize this process to achieve greater control over it [28,29,31].

Subsequent to our data collection and after starting the results analysis for this present research work, some new proposals have been published for possible techniques oriented in this same direction [32]. These proposals underline the frequent error of identifying the "it's like this" derived from experimental studies with "it ought to be done like this" characteristic of CPGs, overlooking the plurality and importance of "internal" conditioning factors not made explicit in the present grading systems.

In general, the proposals published come within the sphere of the so-called "consensus methods" (nominal groups, Delphi techniques etc), more or less modified, and are directed at "controlling" the mentioned "internal dynamics" and making this process more visible. The demands for greater *explicitness *by the experts participating in the research work could be interpreted along these same lines. However, in addition, the influence of these factors is conditioned not only by the interests, priorities and future expectations regarding the issues subject to debate but also those of the guideline developers themselves, and this leads us to the concept of *reflexivity*, a term common to the field of social science and related to making clear the subject of knowledge. This reflexivity means the expert's acceptance of his inevitable active role, not only in the application of a specific procedure, but also in the choice of a particular method to incorporate techniques and instruments directed at selecting "evidence" for its subsequent development into CPG recommendations, and even in identifying the *social and health conditions *that delimit the context in which these choices are made.

As a result of this reflexivity, some standardizable procedures of consensus are unquestionably required to make the dynamics intervening in the CPG development transparent and explicit. These procedures should also address the higher levels of knowledge (methodological and epistemological) [33] responsible for classifying, giving priority to and processing the information on which the CPG elaboration processes are based.

## Conclusion

The aim of this qualitative research work was to gain an insight into the contributions and difficulties perceived by the experts participating in a GRADE method pilot study in the context of its implementability in the Spanish health care system.

The analysis of the findings has allowed us to explain the significance of certain specific problems in the implementation of the GRADE method, some of which had already been detected through a prior questionnaire-based study. These problems would need to be addressed if the GRADE method is to be implemented in Spain.

This work has also revealed the existence of tensions of a more general nature and which affect the CPG consensus and elaboration in the context of the health care sector in Spain. These tensions can be divided into three major areas of debate: a) The need to better adapt the CPG models to the complexity of clinical practice. b) The need for carefully designed multi-disciplinary work to prepare the CPGs and c) The challenges derived from reflexivity in the CPG preparation process.

These lines of discussion point to paths for possible future research directed at gaining a more comprehensive knowledge of the CPG development processes as a particularly important condition for improving the CPG quality.

## Competing interests

The authors declare that they have no competing interests. Mercé Marzo is member of the Grade Working Group. Mercé Marzo, Rafael Rotaeche, Arritxu Etxeberria and Rosa Rico are authors and reviewers of some publications about the Grade System in Spanish journals.

## Authors' contributions

CC and RRo conceived and designed the study. RRo, RRi, AE and MM coordinated the practical development and its diffusion. CC and MB compiled the information. CC carried out the analysis and wrote the paper. All authors reviewed and approved the final manuscript.

## Pre-publication history

The pre-publication history for this paper can be accessed here:



## Figures and Tables

**Table 1:** Participants' profile: academic studies, job and self-referenced experience

**Academic studies and job**	**CPG Experience**
Primary Health Care Pharmacist	Experience in development of recommendations on pharmacological treatments. Experience in Evidence Based Medicine (EBM).
Primary Health Care Pharmacist	EBM Group in Primary Health Care. Coordinator of the Asthma guideline and author of a Hypertension CPG (HBP). Methodological support for CPG developers. Using SIGN method. Workshop organizer.
Family Physician. Osakidetza (Basque Health Service).	EBM Group in Primary Health Care. Coordinator of the HBP guideline and author of a Asthma CPG. Methodological support for CPG developers. Using SIGN method. Workshop organizer.
Family Physician. Primary Health Care Management. Catalan Health Service.	Coordinator of CPG development in a Scientific Society (Semfyc) and Cochrane Collaboration. Using Oxford-CEBM system. Member of the GRADE international group. Workshop organizer.
Family Physician. Osakidetza (Basque Health Service).	EBM Group in Primary Health Care. Experience in the development of a HBP CPG. Using SIGN Method.
Primary Health Care Pharmacist	Experience in the development of a HBP CPG. Using SIGN Method.
Family Physician. Teaching Unit of Family Physician.	EBM Group in Primary Health Care. Back Pain Guideline developer and coordinator. Using SIGN Method.
Family Physician. Osakidetza (Basque Health Service).	EBM Group in Primary Health Care. Back Pain Guideline developer. Using SIGN Method.
Physician.	GUIASALUD Project. (Spanish CPG Clearinghouse)
Primary Health Care Pharmacist.	Participation in the development of a Asthma CPG. Methodological support for CPG developers. Using SIGN method.
Physician. Basque Office for Health Technology Assessment (OSTEBA).	Experience in reviewing scales for assessing quality of evidence.
Physician. OSTEBA.	Experience in carrying out recommendations for Health Technology Assessment Reports. Using the SIGN method as a reference.
Nurse. Osakidetza (Basque Health Service).	EBM Group in a Scientific Society (Osatzen). Experience in the development of a HBP CPG. Using SIGN Method.
Preventive Medicine & Public Health Specialist. OSTEBA.	Experience in carrying out recommendations for Health Technology Assessment Reports. Using the SIGN method as a reference. Methodological support (SIGN, AGREE, ADAPT.) for CPG developers.
Family Physician. Osakidetza (Basque Health Service).	EBM Group in a Scientific Society (Osatzen). Participation in the development of a HBP CPG. Using SIGN method.
Biologist PhD. OSTEBA	Experience in carrying out recommendations for Health Technology Assessment Reports. Using SIGN method as a reference.
Senior Resident of Preventive Medicine & Public Health	Currently carrying out systematic reviews. Using Jovell Scale for qualifying evidence and SIGN for grading recommendations.
Preventive Medicine & Public Health Specialist. PhD. OSTEBA.	Experience in carrying out recommendations for Health Technology Assessment Reports. Methodological support for CPG developers.
Family Physician. Osakidetza (Basque Health Service).	EBM Group in Scientific Society (Osatzen). No experience in CPG development.
Senior Resident of Preventive Medicine & Public Health.	Experience in the development of a Craneo-encephalic Trauma CPG. (SIGN and Oxford CEBM methods).

**Table 2:** Opinion on the GRADE trial through a questionnaire: selection of 5 questions (absolute n° and % over the total number of answers for each section).

Question	Strongly agree	Agree	Neither agree nor disagree	Disagree	Strongly disagree
2. The method is clear and simple to apply.		2 10,5%	4 21,1%	11 57,9%	2 10,5%
4. In general, subjective decisions are unnecessary with the GRADE method.			2 10,5%	12 63,2%	5 26,3%
10. The method successfully discriminates between the different evidence levels.		7 36,8%	8 42,1%	4 21,1%	
11. The method successfully discriminates between the different recommendation levels		11 57,9%	4 21,1%	4 21,1%	
16. The method for grading the recommendation is better than the others you've used		6 31,6%	4 21,1%	8 42,1%	

Source: Formulation of recommendations in Clinical Practice Guidelines: evaluation of the new GRADE system within the Spanish context. 5 AD. Jul 5; Lyon: III GIN International Meeting, 2005 [8].
